# Role of HTRA1 in bone formation and regeneration: *In vitro* and *in vivo* evaluation

**DOI:** 10.1371/journal.pone.0181600

**Published:** 2017-07-21

**Authors:** Gladys Filliat, Ali Mirsaidi, André N. Tiaden, Gisela A. Kuhn, Franz E. Weber, Chio Oka, Peter J. Richards

**Affiliations:** 1 Bone and Stem Cell Research Group, CABMM, University of Zurich, Zurich, Switzerland; 2 Zurich Center for Integrative Human Physiology (ZIHP), University of Zurich, Zurich, Switzerland; 3 Institute for Biomechanics, ETH Zurich, Zurich, Switzerland; 4 Oral Biotechnology & Bioengineering, Center for Dental Medicine, University of Zurich, Zurich, Switzerland; 5 Division of Gene Function in Animals, Nara Institute of Science and Technology, Nara, Japan; Università degli Studi della Campania "Luigi Vanvitelli", ITALY

## Abstract

The role of mammalian high temperature requirement protease A1 (HTRA1) in somatic stem cell differentiation and mineralized matrix formation remains controversial, having been demonstrated to impart either anti- or pro-osteogenic effects, depending on the *in vitro* cell model used. The aim of this study was therefore to further evaluate the role of HTRA1 in regulating the differentiation potential and lineage commitment of murine mesenchymal stem cells *in vitro*, and to assess its influence on bone structure and regeneration *in vivo*. Our results demonstrated that short hairpin RNA-mediated ablation of *Htra1* in the murine mesenchymal cell line C3H10T1/2 increased the expression of several osteogenic gene markers, and significantly enhanced matrix mineralization in response to BMP-2 stimulation. These effects were concomitant with decreases in the expression of chondrogenic gene markers, and increases in adipogenic gene expression and lipid accrual. Despite the profound effects of loss-of-function of HTRA1 on this *in vitro* osteochondral model, these were not reproduced *in vivo*, where bone microarchitecture and regeneration in 16-week-old *Htra1*-knockout mice remained unaltered as compared to wild-type controls. By comparison, analysis of femurs from 52-week-old mice revealed that bone structure was better preserved in *Htra1*-knockout mice than age-matched wild-type controls. These findings therefore provide additional insights into the role played by HTRA1 in regulating mesenchymal stem cell differentiation, and offer opportunities for improving our understanding of how this multifunctional protease may act to influence bone quality.

## Introduction

Mammalian high temperature requirement protease A1 (HTRA1) is one of four HtrA serine protease family members [1, 2], having recently come into prominence by virtue of its predicted involvement in the genetic disorders age-related macular degeneration (AMD) [3, 4] and cerebral autosomal recessive arteriopathy with subcortical infarcts and leukoencephalopathy (CARASIL) [5, 6]. HTRA1, like its three paralogs, contains a trypsin-like protease domain and one PDZ domain [7]. At the amino acid level, HTRA1 shares highest identity with HTRA3 [8]. Furthermore, both HTRA1 and HTRA3 have been detected at comparable locations both in mice and humans [9, 10]. Although primarily regarded as a secreted protease, HTRA1 has been detected in several different subcellular locations [11–13], thus providing alternative routes through which it may influence biological processes. In this regard, HTRA1 has amassed an impressive collection of substrates, including both intracellular (e.g. tuberous sclerosis complex 2, tubulins, tau, and proTGF-β1) [11–14] and extracellular (e.g. bone sialoprotein, fibronectin, elastin, fibromodulin, TGF-β1) [15–19] proteins. Subsequently, interest in HTRA1’s contribution to human development and disease is wide ranging, encompassing numerous research fields such as cancer [20, 21], reproduction [22, 23], neurology [17, 24], and the musculoskeletal system [25].

Findings from our previous studies and from others, have identified HTRA1 as an important factor in determining the lineage commitment of primary mesenchymal stem cells (MSCs), where it acted to promote osteogenesis at the expense of adipogenesis [15, 26–28]. In support of this, HTRA1 protein has been detected in developing bones *in vivo*, as well as in fracture callus [9, 15, 19]. However, in contrast to these findings, several studies now exist in which HTRA1 has been demonstrated to impart a negative influence over osteogenesis [29, 30]. Although the cause of these conflicting results remains unclear, it is important to note that inherent differences exist between the cell culture systems used in each of these studies, and may therefore indicate that cell specific effects of HTRA1 need to be taken into account. In further support of HTRA1’s role in repressing osteogenesis, studies using *Htra1*-knockout mice demonstrated significant improvements in a small number of bone parameters at selected skeletal sites [31]. However, these findings are confounded by the apparent lack of any skeletal aberrations in HTRA1 deficient mice generated by other investigators [32]. Clearly therefore, HTRA1’s regulation of bone formation remains a controversial issue and as such, requires further investigation.

In the current study, we have assessed the role of HTRA1 in regulating osteogenesis *in vitro* and *in vivo*. We determined the effects of loss-of-function of HTRA1 on the differentiation potential of C3H10T1/2 cells stimulated with BMP-2, and on bone development and regeneration in mice. We demonstrated that matrix mineralization was significantly enhanced in HTRA1 deficient C3H10T1/2 cells, in association with the increased expression of several osteogenic gene markers. In addition, adipogenesis was also enhanced in HTRA1 deficient C3H10T1/2 cells, whilst chondrogenic gene expression was downregulated. By contrast, HTRA1 deficiency had no effect on the bone microarchitecture or regeneration of femurs from 16-week-old mice, although bone structure in aged mice was significantly improved as compared to age-matched wild-type controls.

## Materials and methods

### Materials

Human recombinant bone morphogenetic protein-2 (hrBMP-2) was prepared as previously reported [33]. Polyclonal rabbit anti-HTRA1 and anti-HTRA3 were generously provided by Prof. Michael Ehrmann (University of Duisburg-Essen, Germany) [24, 34] and Prof. Chio Oka (NAIST, Japan) [9]. Biotinylated swine anti-rabbit IgG (E0431) was purchased from Dako (Baar, Switzerland).

### Cell culture and differentiation

#### C3H10T1/2 cell line

The murine mesenchymal cell line C3H10T1/2 [35] was kindly provided by Dr. Ronald Biemann (University of Magdeburg, Germany). Cells were cultured in normal growth medium consisting of Dulbecco’s modified eagle medium (DMEM-low glucose, with GlutaMAX; Thermo Fisher Scientific, Reinach, Switzerland), supplemented with 10% foetal bovine serum (FBS; Sigma-Aldrich, Buchs, Switzerland), and penicillin/streptomycin (50 units/ml; 50 μg/ml; Thermo Fisher Scientific). For differentiation studies, cells were seeded at a density of 7’000 cells/cm^2^ and cultured in osteogenic induction medium consisting of normal growth medium supplemented with 10 mM β-glycerophosphate (Sigma-Aldrich), 50 μM L-ascorbic acid 2-phosphate sesquimagnesium salt hydrate (Sigma-Aldrich) and hrBMP-2 (100 ng/ml) for up to 49 days with regular medium changes.

### Lentiviral shRNA

Lentiviral shRNA constructs specific for *Htra1* were purchased from the Sigma Mission library (Sigma-Aldrich) and consisted of TRCN0000031484 (sh*Htra1*^84^) and TRCN0000031486 (sh*Htra1*^86^). The SHC002 non-target control shRNA construct (shControl) was kindly provided by Prof. Michael Ehrmann (University of Duisburg-Essen, Germany). All shRNA construct were cloned into the pLKO.1-puro vector. In order to generate shRNA-expressing lentiviral particles, HEK293T cells were transfected with shRNA plasmids, in combination with packaging plasmid pCD/NL-BH*DDD (Addgene plasmid #17531) [36] and envelope plasmid pLTR-G (Addgene #17532) [37] using calcium phosphate co-precipitation, and lentiviral particles collected at 24 and 48 h. C3H10T1/2 cell cultures were transduced with virus, together with 8 μg/ml polybrene (Sigma-Aldrich), and medium refreshed with normal growth medium after 24 h. Transduced cells were selected for 1 week in the presence of 2 μg/mL puromycin (Sigma Aldrich), and subsequently seeded at 7’000 cells/cm^2^ in cell culture plates.

### RT-qPCR

Reverse-transcription quantitative PCR (RT-qPCR) was performed using TaqMan Gene Expression Assays (Thermo Scientific) (S1 Table) as previously described [27]. Briefly, a total of 0.5 μg of RNA was reverse-transcribed using Superscript II (Thermo Scientific), and successive qPCR reactions performed using the StepOnePlus (Thermo Scientific). Values were normalized to *Rps12* mRNA levels and presented as fold change according to the 2^-ΔΔ*C*T^ method.

### Animals

Mice with targeted mutations in *Htra1* were generated using homologous recombination as previously described [38]. Mice were housed in groups of two to five animals under specific pathogen free conditions, and were allowed to acclimatize for one week prior to surgery. Housing rooms were maintained on a light/dark cycle of 12/12 h with artificial light, and animals were fed a commercial diet and water *ad libitum*. All surgeries were performed under aseptic conditions using isoflurane anaesthesia, and post-operative pain controlled using Buprenorphine. Mice were euthanized by cervical dislocation following isoflurane-induced anaesthesia. All procedures were approved by the Veterinary Office of the Canton of Zurich, Switzerland (Project License 262/2014 and 197/2013) and were carried out in strict accordance with the guidelines of the Swiss Federal Veterinary Office for the use and care of laboratory animals.

### Femoral osteotomy model

A femoral osteotomy model was performed in wild-type (WT) (n = 62) and *Htra1*-knockout (*Htra1*-KO) (n = 61) female mice (16 weeks-of-age) using previously established protocols [39, 40]. The mean weights of WT (22.7 g ± 1.5) and *Htra1*-KO (23.2 g ± 1.8) mice were not significantly different at the time of surgery, and animals were randomly assigned to experimental groups. Mice were injected subcutaneously with Buprenorphine (Temgesic^®^ solution, 0.3 mg/mL; Reckitt Benckiser, Wallisellen, Switzerland) at a dose of 0.1 mg/kg 30 min prior to surgery, and subsequently placed under general anaesthesia using 2% isoflurane and 100% oxygen as a carrier at 400 ml/min. Eye cream was administered to the eyes to prevent drying out. The skin was incised over the lateral aspect of the thigh and a flexible or rigid 4 hole MouseFix plate (RISystem, Davos, Switzerland) secured to the anterolateral aspect of the femur using interlocking screws. A Gigli saw (0.22 mm) was then used to create a mid diaphyseal osteotomy gap with the assistance of a saw guide. The osteotomy site was then irrigated with sterile saline, and the incision closed using Vicryl 6–0 (Ethicon, Norderstedt, Germany) and Appose ULC 35W skin staples (Medtronic, Muenchenbuchsee, Switzerland). Betadine was applied topically to the wound as a preventative measure against possible infection. Post-operative pain was controlled using Buprenorphine (1 mg/kg via drinking water) *ad libitum* for the first 4 days, and animal health and well-being monitored and recorded using a comprehensive scoring system every 12 h for the first 3 days, and then three times per week for the remainder of the study. Anaesthetized mice were euthanized at 10, 14, 21 and 35 days after surgery (n = 8–13 mice/group/time point) by cervical dislocation, and femurs harvested for further analysis.

### Micro-CT analysis of mouse femurs

Following the removal of surrounding soft tissue, mouse femurs were fixed in 4% formaldehyde in phosphate buffered saline (PBS, pH 7.4) for 24 h at 4°C. Bones were then extensively washed in running tap water and stored in 70% ethanol until analysed. Comparisons of bone structure in intact femurs were performed between 16-week-old WT (n = 8), *Htra1*-heterozygous (*Htra1*-HET) (n = 8), and *Htra1*-KO (n = 7) mice; 52-week-old WT (n = 8) and *Htra1*-KO (n = 6) mice. Femurs were scanned on a microCT40 (Scanco Medical AG, Brüttisellen Switzerland) operated at 55 kVp and 145 μA with 200 ms integration time and 2-fold frame averaging. Images were reconstructed from 1000 projections at a nominal isotropic resolution of 10 μm. After application of a Gaussian Filter (sigma 0.8, support 1), image thresholds were set, and automated masks of full bone, cortex and metaphyseal trabecular bone created [41, 42].

Evaluation of bone volume in osteotomy sites stabilized with a flexible MouseFix plate was performed in WT and *Htra1*-KO mice at 21 days (WT, n = 11; *Htra1*-KO, n = 10) and 35 days (WT, n = 10; *Htra1*-KO, n = 13) post-surgery. Analysis of osteotomy sites stabilized with a rigid MouseFix plate was performed in WT and *Htra1*-KO mice at 21 days (WT, n = 9; *Htra1*-KO, n = 9) post-surgery. Following removal of the MouseFix plate, micro-CT measurements were performed using the same settings as the intact femurs described above. After image processing, a threshold of 25% of maximum grey value was applied, and a volume of interest (500 x 500 x 280 voxels) manually selected to accommodate the full callus volume between the inner screws in which the volume of mineralized tissue was calculated (S1 Fig). We chose not to distinguish between original and newly formed bone as no reliable thresholds could be determined. Analysis of osteotomy repair was not performed in cases where the MouseFix plates failed to attach correctly during surgery (WT, n = 6; *Htra1*-KO, n = 7), or showed signs of loosening or dislocation at the time of harvesting (WT, n = 4; *Htra1*-KO, n = 4).

### Histological staining of C3H10T1/2 cell cultures

#### Alizarin Red S

Matrix mineralization was assessed using Alizarin Red S staining as previously described [27]. Cells were washed in phosphate buffered saline (PBS) and fixed in 4% formaldehyde in PBS (pH 7.4) for 1 h at room temperature. Cells were then washed in water and stained with 2% Alizarin Red S (pH 4.2) for 10 min at room temperature. Cells were subsequently washed in PBS, and images captured using a digital camera (Canon EF-S18-55IS2). Alizarin Red S was then extracted in 10% cetylpyridinium chloride (Sigma-Aldrich), and optical densities measured at 570 nm using an Infinite M200 multiplate reader (Tecan) and normalized to cell number as previously described [27].

#### Oil Red O

Lipid accrual was visualized by Oil Red O staining according to previously published protocols [26]. Briefly, cells were washed in PBS and fixed in 4% formaldehyde in PBS (pH 7.4) for 1 h at room temperature. Cells were then rinsed in 60% isopropanol and after drying, stained with 0.3% Oil Red O in isopropanol for 10 min at room temperature. Cells were subsequently washed in water and images captured using a digital camera (Canon EF-S18-55IS2).

### Histological analysis of mouse bone

Mouse femurs were collected at selected time points following osteotomy and fixed in 4% paraformaldehyde in PBS (pH 7.4) for 24 h at 4°C. Bones were subsequently washed in running tap water and incubated in decalcifying solution consisting of 15% ethylenediaminetetraacetic acid (EDTA) (pH 8) with 0.5% paraformaldehyde for up to 2-weeks at 4°C with regular changes [39]. Once decalcified, bones were washed in running tap water overnight, processed and embedded in paraffin wax.

#### Safranin O/ Fast Green

Safranin O/ Fast Green staining was used to visualize cartilaginous callus formation in paraffin wax sections (8 μm) of femurs at 10, 14 and 21 days following osteotomy. Tissue sections were dewaxed, rehydrated and stained in 0.05% Fast Green for 5 min. Slides were directly transferred to 0.1% Safranin O for 5 min and following dehydration in graded alcohols, mounted in DPX. Images were captured using a Leica M205C stereo microscope (Leica Microsystems, Heerbrugg, Switzerland) fitted with a digital camera (Canon EF-S18-55IS2). The ratio of Safranin O positive area to callus area was determined using NIH ImageJ software, where at least three serial tissue sections of the central callus region between the inner screws from 7 to 10 mice per group were analysed.

#### Immunohistochemistry

Tissue sections were dewaxed, rehydrated and treated sequentially with Avidin/Biotin Blocking Kit (Abcam), 3% H_2_O_2_ and normal swine serum (Reactolab, Servion, Switzerland) to reduce non-specific staining. Sections were then incubated for 1 h at 37°C with either polyclonal rabbit anti-HTRA1 (1:300), polyclonal rabbit anti-HTRA3 (1:400), or equivalent dilutions of normal serum. After washing in PBS, sections were incubated with a biotinylated swine anti-rabbit IgG (1:400) for 45 min at 37°C followed by washing and a further incubation for 30 min with Vectastain (Reactolab). Sections were developed using 3,3' diaminobenzidine tetrahydrochloride (DAB) (Sigma-Aldrich), counterstained with Harris modified hematoxylin (Sigma-Aldrich) and visualized using an Olympus BX51 light microscope (Olympus Schweiz AG, Volketswil, Switzerland).

### Statistical analysis

Two-tailed unpaired Student’s *t*-test was used for comparison of two groups, and one-way analysis of variance (ANOVA) with Tukey’s post hoc test was used for multiple group comparisons. In all cases, a *P*-value of < 0.05 was considered statistically significant, and all data were expressed as mean ± standard deviation (S.D.).

## Results

### Loss-of-function of HTRA1 enhances osteogenic differentiation of C3H10T1/2 cells

**Fig 1:**
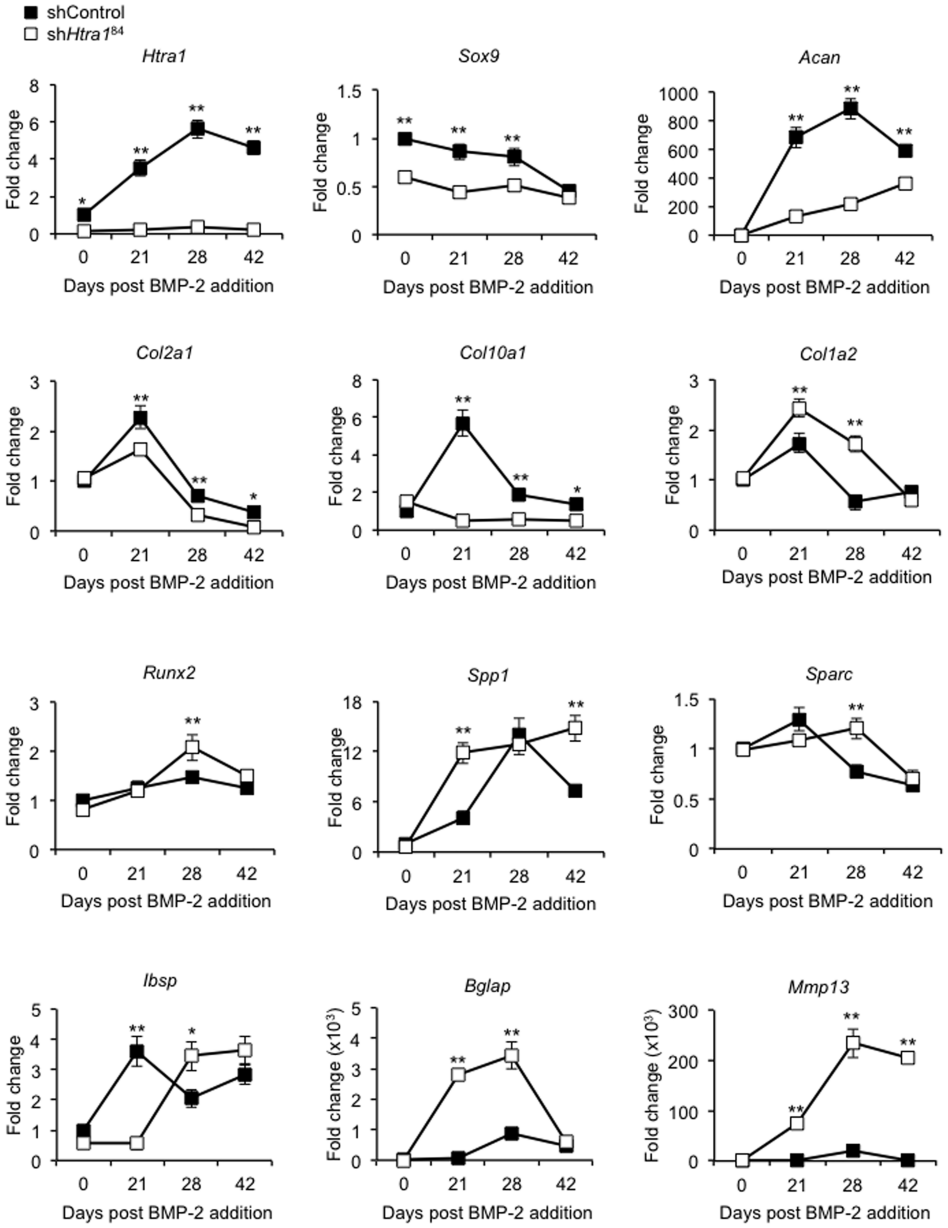
Effect of *Htra1* knockdown on gene expression in BMP-2 stimulated C3H10T1/2 cells. RT-qPCR analysis was used to determine the expression levels of *Htra1*, *Sox9*, *Acan*, *Col2a1*, *Col10a1*, *Col1a2*, *Runx2*, *Spp1*, *Sparc*, *Ibsp*, *Bglap* and *Mmp13* in C3H10T1/2 cells transduced with non-target control shRNA (shControl) or *Htra1*-specific shRNA (sh*Htra1*^84^) at selected time points following stimulation with rhBMP-2 (100 ng/ml). Gene expression levels were determined using the 2^-ΔΔ*C*T^ method and presented as fold change relative to uninduced cells at day 0 (value = 1). All values are expressed as mean ± S.D. (triplicates). *P < 0.05, **P < 0.01 comparison between shControl and sh*HtrA1*^84^ using one-way ANOVA.

The multipotent mouse cell line C3H10T1/2 represents a useful tool for investigating osteogenesis *in vitro*, having the capability of simulating many of the characteristics associated with endochondral ossification in response to BMP-2 stimulation [43, 44]. In the current report, we used lentivirus-delivered shRNA targeting the *Htra1* gene to assess the influence of loss-of-function of HTRA1 on BMP-2-induced osteochondral differentiation of C3H10T1/2 cells over the course of 7 weeks. We observed a time dependent increase in *Htra1* expression in response to BMP-2 in C3H10T1/2 cells treated with shRNA control vector (shControl), reaching a maximum of 5.6-fold (± 0.5) at day 28 (Fig 1). Attempts were also made to measure *Htra3* and *Htra4* expression levels, but values remained below detectable limits. Transduction of C3H10T1/2 cells with *Htra1*-specific shRNA (sh*Htra1*^84^) efficiently suppressed *Htra1* gene expression throughout the course of the study, and significantly altered the temporal gene expression profiles of selected chondrocyte and osteogenic markers in response to BMP-2 stimulation. Expression levels of the chondrogenic markers *Sox9*, *Acan*, *Col2a1* and *Col10a1* were significantly reduced in sh*Htra1*^84^-treated cells as compared to shControl-treated cells at the majority of time points tested. By contrast, the expression levels of several osteogenic markers including *Col1a2*, *Runx2*, *Spp1*, *Sparc*, and most notably, *Bglap* and *Mmp13*, were significantly enhanced at selected time points in HTRA1 deficient cells. Interestingly, BMP-2 induced *Ibsp* expression appeared to be delayed in HTRA1 deficient cells, and was significantly lower than shControl-treated cells at day 21. However, by day 28, *Ibsp* expression levels in sh*Htra1*^84^-treated cells had increased, and were significantly enhanced as compared to shControl cells.

**Fig 2:**
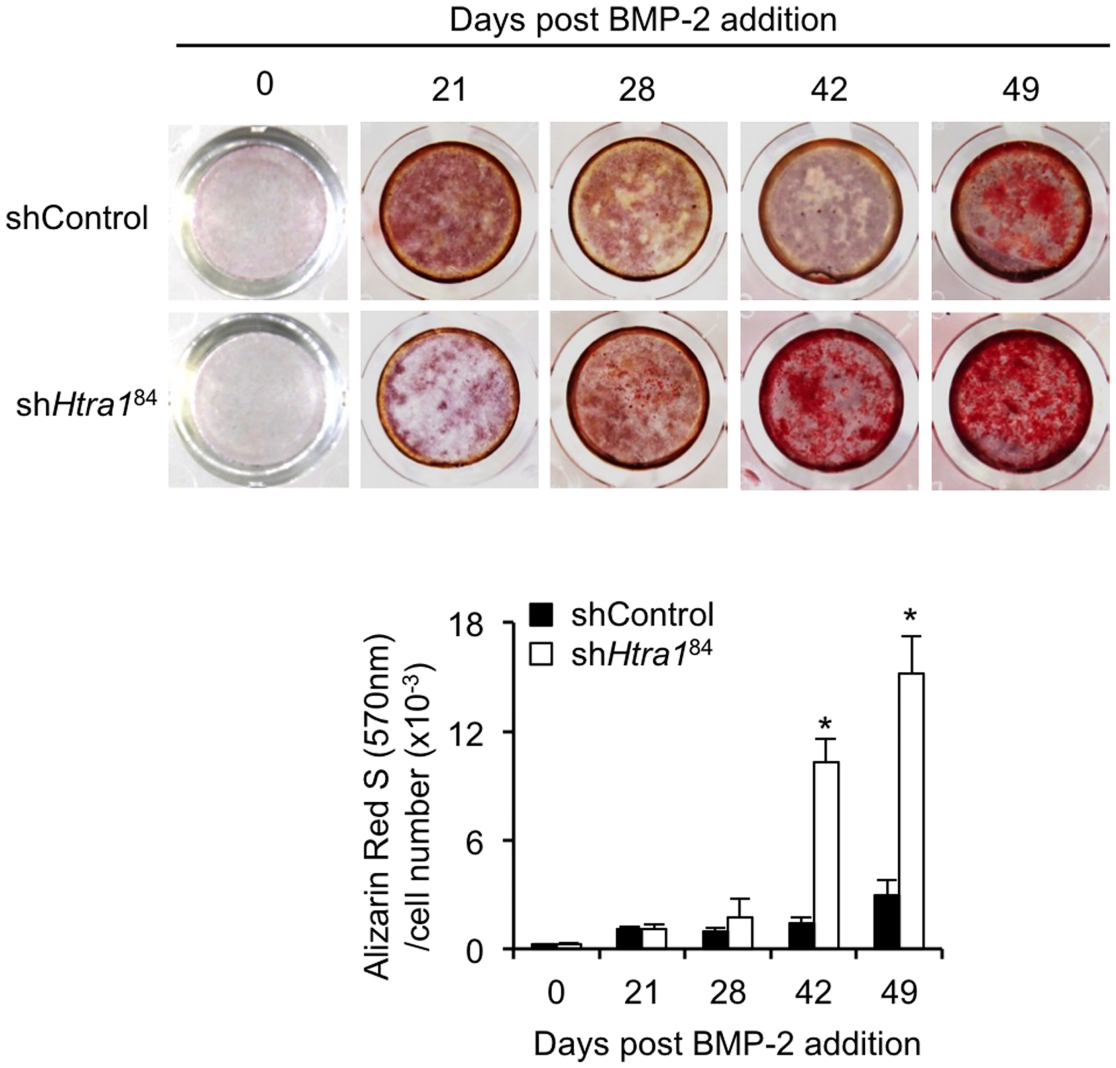
Effect of *Htra1* knockdown on mineralized matrix production in BMP-2 stimulated C3H10T1/2 cell cultures. CH310T1/2 cells stably transduced with non-target control shRNA (shControl) or *Htra1*-specific shRNA (sh*Htra1*^84^) were stimulated with rhBMP-2 (100 ng/ml) for up to 49 days and stained with Alizarin Red S. The extracted dye was quantified and normalized to cell number. All values are expressed as mean ± S.D. (triplicates). *P < 0.01, as compared to shControl using one-way ANOVA.

It therefore appeared that loss of HTRA1 favoured a more osteogenic lineage commitment of C3H10T1/2 cells in response to BMP-2. In accordance with this, *Htra1* knockdown also significantly enhanced mineralized matrix deposition at day 42 and 49 as determined by Alizarin Red S staining (Fig 2). Similar effects were also observed when C3H10T1/2 cells were transduced with an alternative *Htra1*-specific shRNA oligonucleotide (sh*Htra1*^86^) (S2 Fig). These data therefore confirm that HTRA1 loss acts to promote C3H10T1/2 osteogenesis and matrix mineralization.

**Fig 3:**
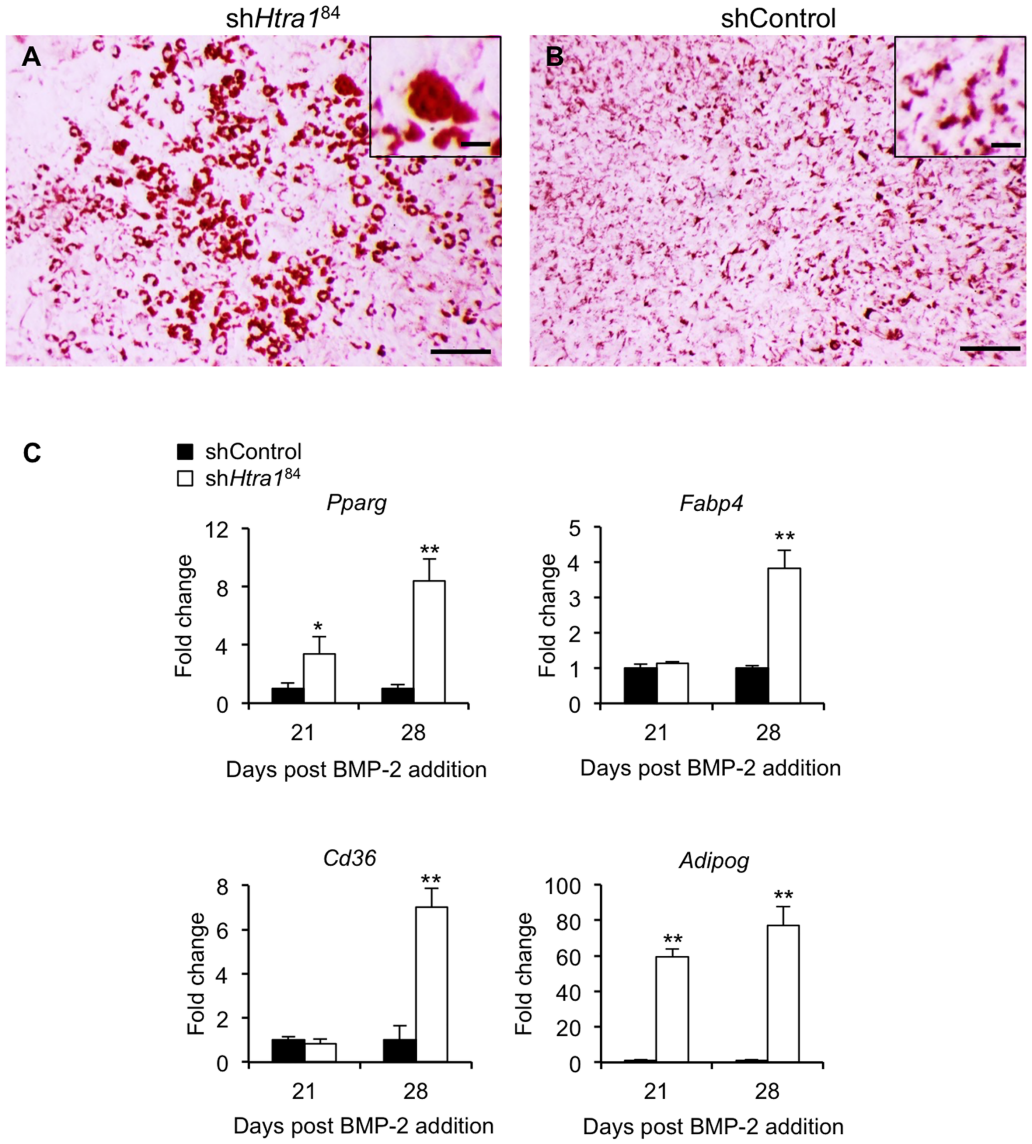
*Htra1* knockdown enhances C3H10T1/2 adipogenesis. CH310T1/2 cells stably transduced with *Htra1*-specific shRNA (sh*Htra1*^84^) (A) or non-target control shRNA (shControl) (B) were stimulated with rhBMP-2 (100 ng/ml) for 23 days and stained with Oil Red O. Scale bar = 500 μm; inset scale bar = 25 μm. (C) RT-qPCR was used to measure expression levels of *Pparg*, *Fabp4*, *Cd36* and *Adipog* after 21 and 28 days stimulation with rhBMP-2 (100 ng/ml). All values are expressed as mean ± S.D. (triplicates). *P < 0.05, **P < 0.01 comparison between shControl and sh*HtrA1*^84^ using Student’s *t*-test.

During the course of these studies, we noticed what appeared to be adipocytes present within C3H10T1/2 cell cultures treated with sh*Htra1*^84^ following 3 to 4 weeks of stimulation with BMP-2. Indeed, Oil Red O staining confirmed the presence of numerous lipid laden cells in C3H10T1/2 cultures transduced with sh*Htra1*^84^ (Fig 3A) as compared to those transduced with shControl (Fig 3B). Furthermore, expression levels of several adipogenic markers including *Pparg*, *Fabp4*, *Cd36* and *Adipog* were significantly increased in sh*Htra1*^84^-treated cells (Fig 3C). These results therefore demonstrated that the stimulatory effects of loss-of-function of HTRA1 on C3H10T1/2 osteogenesis were paralleled by increases in adipocyte formation.

### Bone structure and regeneration are unaffected in 16-week-old HTRA1-deficient mice

**Fig 4:**
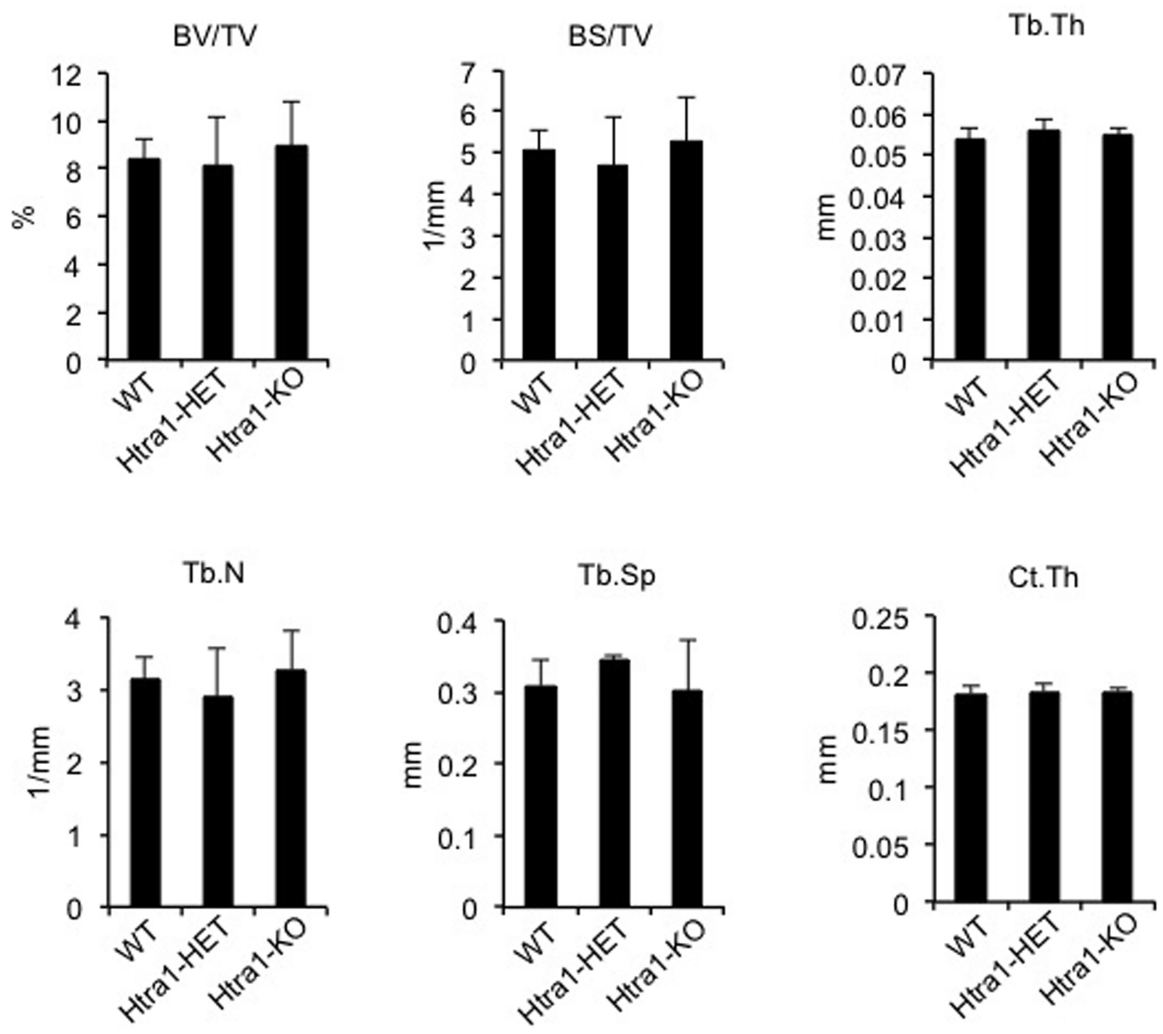
Mouse femur microstructure is unaffected by HTRA1 deficiency. Micro-CT analysis of femurs from 16-week-old wild-type (WT, n = 8), *Htra1*-heterozygous (*Htra1*-HET, n = 8), and *Htra1*-knockout (*Htra1*-KO, n = 7) mice. BV/TV, trabecular bone volume fraction; BS/TV, trabecular bone surface density; Tb.Th, trabecular thickness; Tb.N, trabecular number; Tb.Sp, trabecular spacing; Ct.Th, cortical thickness. All results are expressed as mean ± S.D.

Having identified HTRA1 as a mediator of osteochondral differentiation *in vitro*, we next asked the question whether these effects were also apparent *in vivo*. In order to investigate this, we used a well established *Htra1*-null mouse strain generated through targeted mutation of exon 1 [22, 38]. Unexpectedly, we failed to identify any significant differences in trabecular or cortical bone structure between the femurs of wild-type (WT), heterozygous (*Htra1*-HET) and homozygous (*Htra1*-KO) *Htra1*-knockout mice, as determined by micro-CT (Fig 4). Therefore, it appeared that normal bone development, at least in mice, was not dependent on functional HTRA1.

**Fig 5:**
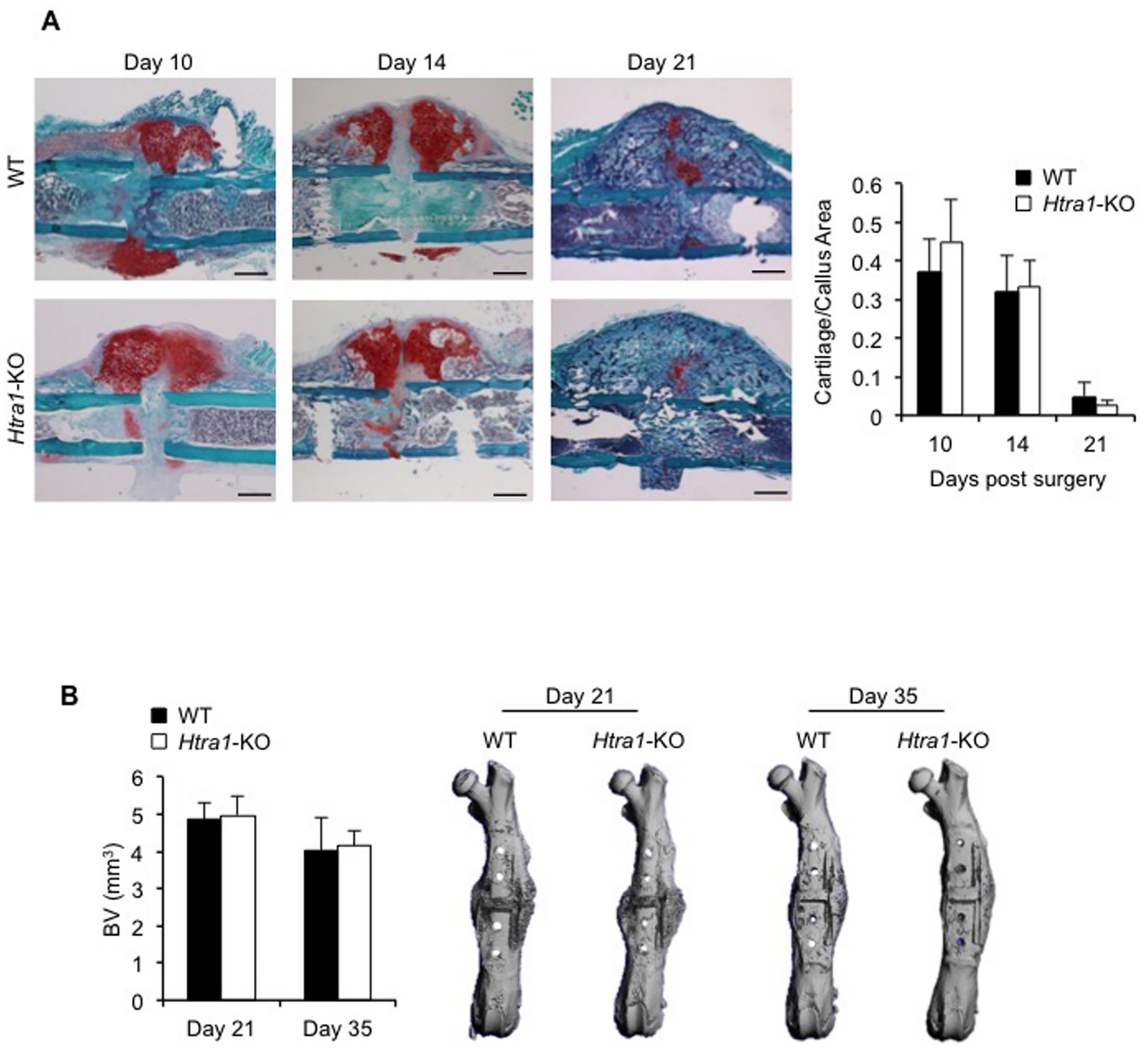
Bone repair in mice is unaffected by HTRA1 deficiency. (A) Cartilage area within osteotomy calluses of wild-type (WT) and *Htra1*-knockout (*Htra1*-KO) mice was determined by Safranin O staining (*red*) at 10 days (WT, n = 8; *Htra1*-KO, n = 9), 14 days (WT, n = 10; *Htra1*-KO, n = 7), and 21 days (WT, n = 9; *Htra1*-KO, n = 7) after surgery. (B) Micro-CT evaluation of bone volume (BV) in osteotomy sites of femurs stabilized with a flexible MouseFix plate from wild-type (WT) and *Htra1*-knockout (*Htra1*-KO) mice at 21 days (WT, n = 11; *Htra1*-KO, n = 10) and 35 days (WT, n = 10; *Htra1*-KO, n = 13) after femoral osteotomy. All results are expressed as mean ± S.D.

As with long bone development, fracture healing involves a well coordinated series of events mediated, in part, through the actions of chondroprogenitor and osteoprogenitor cells, culminating in the production and eventual mineralization of a hyaline cartilage matrix [45]. However, in contrast to skeletal development, bone repair relies heavily on inflammatory cues to generate the prerequisite mesenchymal stem cell condensations responsible for initiating the cartilaginous template and subsequent mineralized matrix [46]. Clearly, therefore, important differences exist between bone development and repair, which could act to influence HTRA1’s impact on new bone formation. With this in mind, we next proceeded to investigate the effects of HTRA1 ablation on bone repair. An osteotomy defect was generated in the femurs of WT and *Htra1*-KO mice, and stabilized with a flexible MouseFix plate in order to promote endochondral ossification, and thus allow for the visualization of cartilage and bone formation. Surgical intervention was well tolerated by both mouse strains, with no adverse events observed, and weight loss remaining within acceptable limits (< 15% total body weight). Histological analysis of paraffin wax tissue sections using Safranin O/ Fast green demonstrated comparable amounts of cartilage callus in the osteotomy defects of WT and *Htra1*-knockout mice at days 10, 14 and 21 (Fig 5A). Similarly, analysis of osteotomy defect sites at days 21 and 35 using micro-CT identified comparable amounts of mineralized bone between both mouse strains (Fig 5B). Additional osteotomy studies were also undertaken using a rigid MouseFix plate in order to determine whether HTRA1 loss affected bone repair under conditions more conducive to intramembranous ossification. However, bone volume within rigid stabilized osteotomy sites was also found to be comparable between both mouse strains, indicating that the lack of deviations in bone regeneration in *Htra1*-KO mice was not due to the method of osteotomy stabilization (S3 Fig).

**Fig 6:**
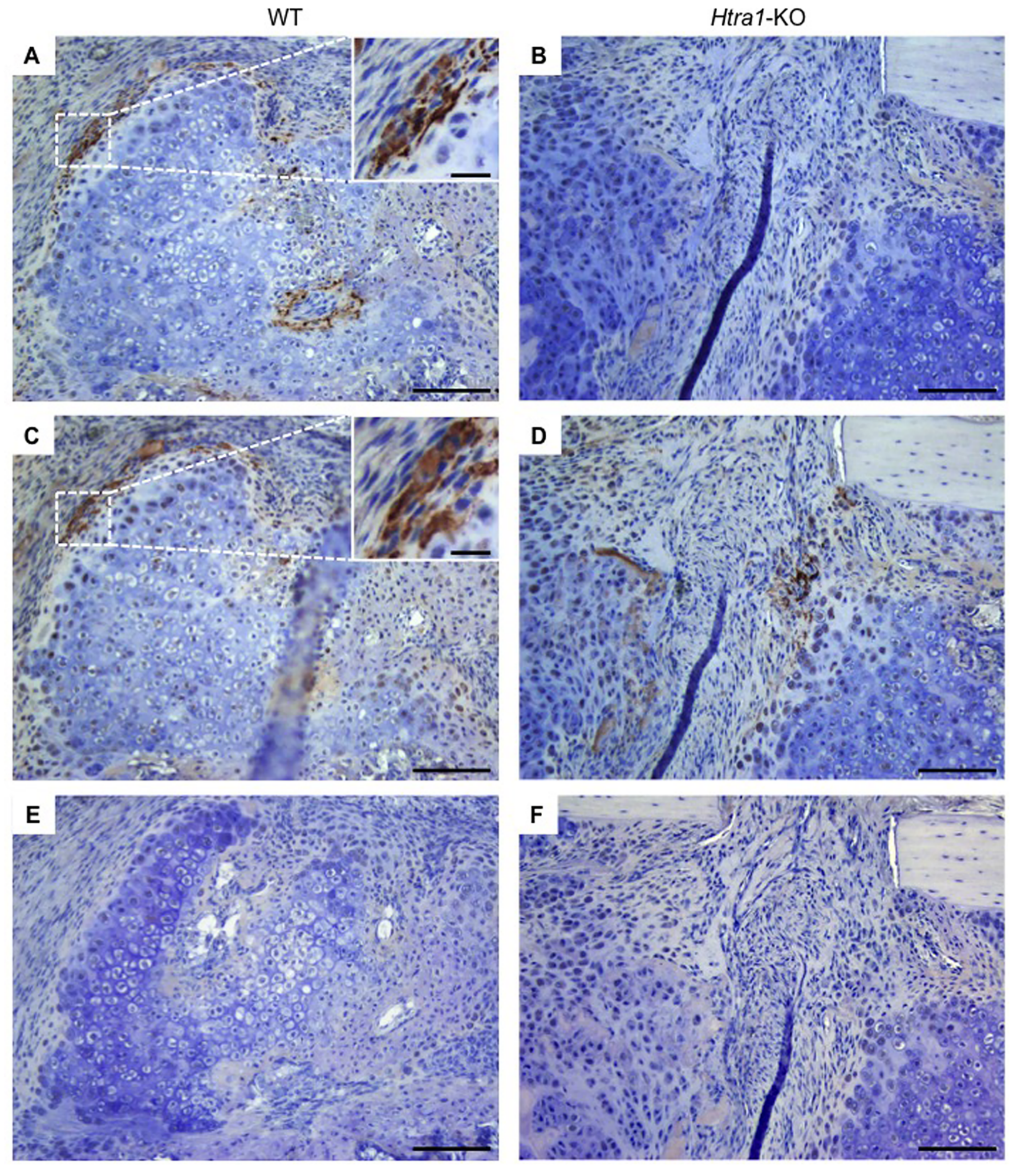
Immunostaining of HTRA1 and HTRA3 in callus tissue. Representative micrographs of anti-HTRA1 (A, B), anti-HTRA3 (C, D), or normal rabbit serum (E, F) stained paraffin wax sections of femurs from WT (A, C, E) and *Htra1*-KO (B, D, F) mice 14 days after osteotomy. HTRA1 and HTRA3 staining was detected using horseradish peroxidase-diaminobenzidine (*brown*) and sections counterstained with Harris modified hematoxylin (*blue*). Main scale bar = 100 μm; inset scale bar = 20 μm.

Immunohistological analysis of tissue sections of the osteotomy site in *Htra1*-KO mice revealed positive staining for HTRA1 protein predominantly in cells surrounding the callus cartilage at day 14 (Fig 6A), as well as in chondro-osseous transition zones at day 21 (S4A Fig). As anticipated, HTRA1 protein was not detected in any of the tissue sections analysed from *Htra1*-KO mice (Fig 6B and S4B Fig). Based on the close structural, and potentially functional similarities between HTRA1 and HTRA3 [8], we also assessed the expression of HTRA3 in osteotomy sites. Indeed, we could detect HTRA3 protein at day 14 and day 21 in sections from WT (Fig 6C and S4C Fig) and *Htra1*-KO mice (Fig 6D and S4D Fig). Furthermore, HTRA1 and HTRA3 were detected at comparable locations in the osteotomy sites of WT mice, and in some cases were even expressed by the same cell populations, thereby suggesting possible overlapping functions (inset Fig 6A and 6C; inset S4A and S4C Fig).

### HTRA1 influences the aging bone phenotype

**Fig 7:**
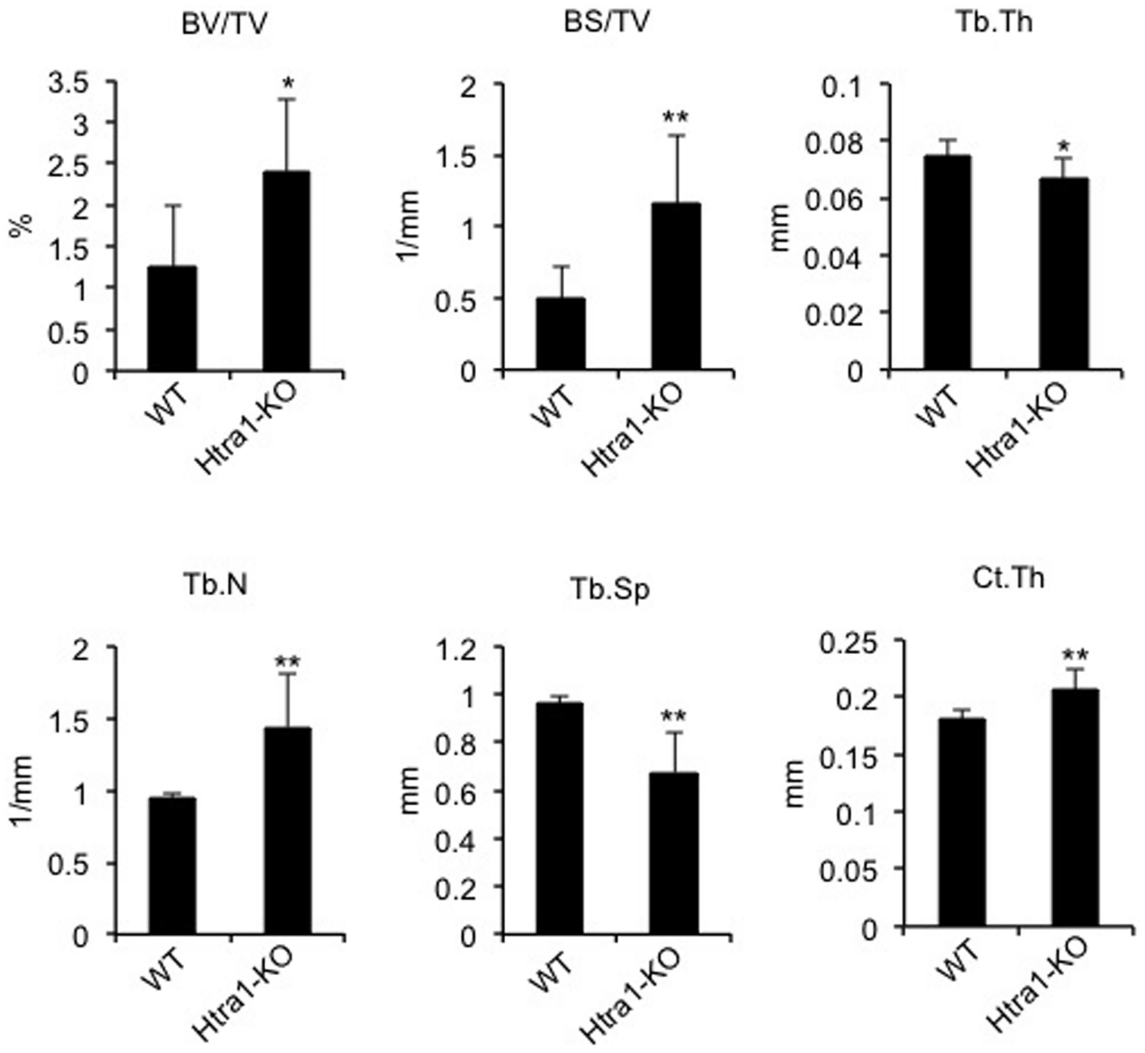
Femur microstructure is improved in HTRA1-deficienct mice. Micro-CT analysis of femurs from 52-week-old wild-type (WT, n = 8) and *Htra1*-knockout (*Htra1*-KO, n = 6) mice. BV/TV, trabecular bone volume fraction; BS/TV, trabecular bone surface density; Tb.Th, trabecular thickness; Tb.N, trabecular number; Tb.Sp, trabecular spacing; Ct.Th, cortical thickness. All results are expressed as mean ± S.D. *P < 0.05, **P < 0.01 using Student’s *t*-test.

It has previously been demonstrated that mouse embryonic fibroblasts harvested from *Htra1*-KO mice are more resistant to premature cell senescence than wild-types [47], thereby providing a possible link between HTRA1 activity and age-related processes. This is further supported by the recent finding that significant increases in systemic levels of HTRA1 were associated with increased incidences of frailty in elderly patients [48]. We therefore asked the question whether aging had any influence on the bone phenotype of *Htra1*-KO mice. Indeed, micro-CT analysis demonstrated significant improvements in trabecular and cortical parameters of femurs from 52-week-old *Htra1*-KO mice as compared to age-matched wild-type mice (Fig 7 and S2 Table and S5 Fig). Although significant increases in trabecular thickness were observed in wild-type mice, trabecular spacing was found to be significantly lower in *Htra1*-KO mice, thereby indicating that the improved trabecular spacing in these mice was primarily due to increases in trabecular number.

## Discussion

Despite several studies having identified HTRA1 in calcified tissue, its actual role in bone formation continues to remain an enigma. In the current report, we set out to evaluate the effects of long-term HTRA1 depletion on bone formation both *in vitro* and *in vivo*. Our findings demonstrated that although HTRA1 deficiency had a profound effect on osteogenic lineage commitment *in vitro*, it failed to influence bone structure and regeneration when assessed in 16-week-old mice. Interestingly however, significant improvements in various structural bone parameters were observed in 52-week-old HTRA1-deficient mice as compared to age-matched wild-type controls, thereby providing a possible link between HTRA1 expression and the bone aging phenotype.

Indications that HTRA proteins may play a role in regulating bone formation initially came from developmental studies performed in mice, where *in situ* hybridization and immunohistochemical analyses identified HTRA1 and HTRA3 within bone tissue [9, 19, 49]. However, it wasn’t until several years later that evidence emerged of a possible functional role for HTRA1 in bone formation. Studies performed using mouse-derived osteoblasts demonstrated that despite its upregulation in response to BMP-2, HTRA1 acted as a negative regulator of bone formation [29, 30]. Moreover, the observation that *Htra1*-deficient mice have a moderately improved bone phenotype [31], suggested that HTRA1’s influence on bone may go beyond simply affecting mineral formation *in vitro*. However, an equal number of studies also now exist in which HTRA1 has been shown to have a positive influence on matrix mineralization *in vitro*. We and others have previously reported that loss-of-function of HTRA1 in primary human and mouse MSCs results in impaired osteogenic differentiation [15, 27, 28]. Furthermore, the overexpression of HTRA1, or its exogenous addition, had the capacity to significantly enhance matrix mineralization by MSC-derived osteoblasts [15, 28]. A major difference between these studies and those in which HTRA1 was reported to negatively influence osteogenesis, is that primary MSCs were used as opposed to immortalized cell lines (2T3 cells) or long-term bone marrow cultures (KusaO cells). As such, HTRA1’s potential to modify osteogenic differentiation and bone mineral formation may be dependent on cell type.

In the current report, we have extended these investigations to include the murine mesenchymal cell line C3H10T1/2. In contrast to our previous findings, we demonstrated that loss-of-function of HTRA1 promoted C3H10T1/2 osteogenic differentiation and matrix mineralization. One possible explanation for these conflicting results is provided by the observation that HTRA1-deficient C3H10T1/2 cells had a significantly greater tendency to undergo adipogenesis. In stark contrast to primary MSCs, including those derived from fat [50] and bone [51], the osteogenic induction of C3H10T1/2 cells is positively regulated by pro-adipogenic gene expression [52]. Therefore, it’s quite possible that the stimulatory effects of HTRA1 deficiency on C3H10T1/2 osteogenesis were indirectly due to increases in the expression of adipogenic gene markers. The concept of loss-of-function of HTRA1 favouring adipogenesis has previously been demonstrated in human BMSCs [15, 26], although unlike HTRA1-deficient C3H10T1/2 cells, this resulted in significant reductions in their osteogenic potential [15]. Currently, it’s unclear whether HTRA1-deficient C3H10T1/2 cells represent a heterogeneous population of adipocytes and osteoblasts, or if they are one in the same. Certainly, evidence does exist to suggest that MSC-derived adipocytes have the potential to undergo transdifferentiation into osteoblasts, and even chondrocytes [53]. Taken together, these results further exemplify the complexities involved in trying to decipher HTRA1’s role in bone formation, and provide additional support for the concept that its effects on osteogenic differentiation are predominantly cell-type specific.

In order to better understand the implications of HTRA1 loss on the physiology of bone formation, several *Htra1*-knockout mouse models have been generated by different research groups. However, as with the findings from studies evaluating the effects of HTRA1 loss *in vitro*, results emanating from these *in vivo* investigations also appear to be beset by inconsistencies. Graham *et al* (2013) observed improvements in various bone structural parameters in *Htra1*-knockout mice as determined by micro-CT, and concluded that this was most likely due to enhanced TGF-β signaling based on the fact that HTRA1 could cleave TGF-β receptors [31]. However, it should be pointed out that in a more recent study by Beaufort *et al* [54], HTRA1 was shown to positively regulate TGF-β pathway activation *in vivo*, although its influence on bone structure was not reported. Results from our own micro-CT analysis of intact femurs taken from 16-week-old *Htra1*-knockout mice demonstrated their bone structure to be comparable to that of age-matched wild-type mice. Moreover, despite confirming the presence of HTRA1 protein within regenerating bone of wild-type mice, and its absence from *Htra1*-knockout mouse tissue, bone repair also appeared to be unaffected by the loss of HTRA1. HTRA1’s localization to, and potential involvement in, new bone formation has previously been attested to in a recent *in vivo* study examining the effects of thyroxine exposure on calvarial growth sites in mice, where enhanced levels of HTRA1 were identified at sites of increased osteoblast activity [55]. We were therefore surprised not to have observed any significant deviations in bone regeneration in HTRA1-deficient mice. Interestingly, immunohistochemical staining of regenerating bone also detected HTRA3 at similar locations as HTRA1 in wild-type mice, as well as in the callus of *Htra1*-knockout mice. As far as we are aware, this is the first report of HTRA3 within bone tissue of adult mice undergoing bone repair. As with HTRA1, it was primarily detected at the borders of cartilaginous tissue within the callus, where chondrocyte apoptosis and subsequent bone remodelling are thought to occur [56]. These findings therefore signify possible functional redundancy between these two HtrA paralogs, whereby loss of HTRA1 is compensated for by HTRA3. Further investigations into bone regeneration using mice deficient in HTRA3, or HTRA1 and HTRA3, may help to provide additional insights into the role of HtrA proteases in bone formation.

Previous studies have identified HTRA1 as an inducer of premature cell senescence [47], and elevated levels of HTRA1 have been positively correlated with increased incidences of frailty in the aged [48]. Therefore, we also considered the possibility that changes in the bone phenotype of *Htra1*-knockout mice may become more apparent with aging. Indeed, the bone structure of femurs from 52-week-old mice was significantly improved in *Htra1*-knockout mice as compared to their wild-type counterparts. These findings therefore suggest that, in mice at least, HTRA1 may represent an important determining factor for bone quality in response to aging, and further studies examining bone regeneration in aged HTRA1-deficient mice may be warranted. Certainly, these new findings are more in keeping with our *in vitro* data, where mineralized matrix formation was enhanced in HTRA1-deficient C3H10T1/2 cells. However, there still exists the matter of reconciling these observations with the results obtained from previous studies investigating the effects of loss or gain of HTRA1 function on MSC lineage commitment [15, 26–28]. The choice of cell type, and preference for immortalized cell line over primary cells, may have played some part in defining HTRA1 as a pro- or anti-osteogenic mediator. Certainly, the response of cultured cells to loss of HTRA1 varies considerably, where for instance proliferation is either decreased [57], enhanced [58], or unaffected [59] depending on the cell source used. Therefore, some caution should be taken in translating these *in vitro* findings to an *in vivo* system, where the generation of a particular phenotype may culminate from a series of heterogeneous cellular responses to alterations in HTRA1 production. Taken together, our findings further identify HTRA1 as a potent regulator of the multilineage differentiation potential of MSCs, and provide evidence to suggest that although HTRA1 does not appear to influence bone development and regeneration beyond the *in vitro* system, it may contribute to the aging bone phenotype in mice. Whether this also applies to aged human bone, however, remains to be determined.

## Supporting information

S1 ARRIVE Checklist NC3Rs ARRIVE guidelines checklist. (PDF)

S1 Table List of TaqMan gene expression assays used in RT-qPCR analysis. (DOCX)

S2 Table Full list of bone morphometric indices used in micro-CT analysis of femurs from 52-week-old mice. (DOCX)

S1 Fig Micro-CT analyses of bone within osteotomy site of mouse femur. Representative images of posterior, anterior, lateral and medial aspects of an *Htra1*-KO mouse femur at 21 days following osteotomy. The red colouration highlights the mineralized tissue within the volume of interest (500 x 500 x 280 voxels) as observed following removal of the MouseFix plate.
(TIF)

S2 Fig Effect of *Htra1* knockdown on mineralized matrix production in BMP-2 stimulated C3H10T1/2 cell cultures. (A) RT-qPCR analysis was used to confirm efficient knockdown of *Htra1* gene expression in C3H10T1/2 cells transduced with *Htra1*-specific shRNA (sh*Htra1*^86^) at selected time points following stimulation with rhBMP-2 (100 ng/ml). Gene expression levels were determined using the 2^-ΔΔ*C*T^ method and presented as fold change relative to uninduced cells at day 0 (value = 1). All values are expressed as mean ± S.D. (triplicates). *P < 0.01 comparison between shControl and sh*Htra1*^86^ using one-way ANOVA. (B) CH310T1/2 cells stably transduced with non-target control shRNA (shControl) or *Htra1*-specific shRNA (sh*Htra1*^86^) were stimulated with rhBMP-2 (100 ng/ml) for up to 42 days and stained with Alizarin Red S.
(TIF)

S3 Fig Bone repair in mice using a rigid MouseFix plate. Micro-CT evaluation of bone volume (BV) in osteotomy sites of femurs stabilized with a rigid MouseFix plate from wild-type (WT) (n = 9) and *Htra1*-knockout (*Htra1*-KO) (n = 9) mice at 21 days after femoral osteotomy. All values are expressed as mean ± S.D.
(TIF)

S4 Fig Immunostaining of HTRA1 and HTRA3 in callus tissue at day 21. Representative micrographs of anti-HTRA1 (A, B), anti-HTRA3 (C, D), or normal rabbit serum (E, F) stained paraffin wax sections of femurs from WT (A, C, E) and *Htra1*-KO (B, D, F) mice 21 days after osteotomy. HTRA1 and HTRA3 staining was detected using horseradish peroxidase-diaminobenzidine (*brown*) and sections counterstained with Harris modified hematoxylin (*blue*). Main scale bar = 50 μm; inset scale bar = 25 μm.
(TIF)

S5 Fig Micro-CT analyses of femurs from 52-week-old WT and *Htra1*-KO mice. Selected images of distal femurs from wild-type (WT) and *Htra1*-knockout (*Htra1*-KO) mice illustrating the regions from which cortical (orange) and trabecular (red) bone measurements were taken. Images are representative of the median trabecular BV/TV value from each group.
(TIF)
