# Supplementary material for: Role of HTRA1 in bone formation and regeneration: In vitro and in vivo evaluation
Source: PLoS One. 2017 Jul 21;12(7):e0181600. doi: 10.1371/journal.pone.0181600 (PMC5521800; doi:10.1371/journal.pone.0181600)
Supplement: S1 Table — (DOCX) [file pone.0181600.s002.docx]

**S1 Table. List of TaqMan Gene Expression Assays used in RT-qPCR analysis**

| **Gene Symbol** | **Protein Product** | **Assay ID *^a^*** |
| --- | --- | --- |
| *Htra1* | High temperature requirement protease A1 | Mm00479887_m1 |
| *Htra3* | High temperature requirement protease A3 | Mm00472631_m1 |
| *Htra4* | High temperature requirement protease A4 | Mm01210984_m1 |
| *Sox9* | SRY-homeobox-like gene 9 | Mm00448840_m1 |
| *Acan* | Aggrecan | Mm00545794_m1 |
| *Col1a2* | Collagen type I, alpha 2 | Mm00483888_m1 |
| *Col2a1* | Collagen type II, alpha 1 | Mm01309565_m1 |
| *Col10a1* | Collagen type X, alpha 1 | Mm00487041_m1 |
| *Runx2* | Runt-related transcription factor 2 | Mm00501584_m1 |
| *Spp1* | Secreted phosphoprotein 1 | Mm01611440_mH |
| *Sparc* | Secreted protein acidic and rich in cysteine | Mm00486332_m1 |
| *Ibsp* | Integrin Binding Sialoprotein | Mm00492555_m1 |
| *Bglap* | Bone gamma-carboxyglutamate (gla) protein | Mm03413826_m1 |
| *Mmp13* | Matrix metalloproteinase 13 | Mm00439491_m1 |
| *Cd36* | Cluster of differentiation 36 | Mm00432398_m1 |
| *Fabp4* | Fatty acid binding protein 4 | Mm00445878_m1 |
| *Pparg* | Peroxisome proliferator activated receptor γ | Mm00440940_m1 |
| *Adipog* | Adiponectin | Mm00456425_m1 |
| *Mrps12* | Mitochondrial ribosomal protein S12 | Mm00488728_m1 |

^a^TaqMan Expression Assay identity code according to supplier (Thermo Fisher

Scientific, Reinach, Switzerland).
