# Supplementary material for: Role of HTRA1 in bone formation and regeneration: In vitro and in vivo evaluation
Source: PLoS One. 2017 Jul 21;12(7):e0181600. doi: 10.1371/journal.pone.0181600 (PMC5521800; doi:10.1371/journal.pone.0181600)
Supplement: S2 Table — (DOCX) [file pone.0181600.s003.docx]

**S2 Table. Full list of bone morphometric indices used in micro-CT analysis of femurs from 52-week-old mice.**

| **Index** | **WT (N=8)** | ***Htra1*-KO (N=6)** | ***P*-value*^a^*** |
| --- | --- | --- | --- |
| AVD (%) | 45.36 (± 0.34) | 47.78 (± 2.38) | 0.018 |
| BV/TV (%) | 1.25 (± 0.74) | 2.4 (± 0.85) | 0.019 |
| BS/TV (1/mm) | 0.49 (± 0.23) | 1.16 (± 0.49) | 0.005 |
| BS/BV (1/mm) | 41.16 (± 3.69) | 47.46 (± 6.91) | 0.036 |
| Tb.Th (mm) | 0.0745 (± 0.005) | 0.0665 (± 0.007) | 0.034 |
| Tb.Sp (mm) | 0.96 (± 0.03) | 0.675 (± 0.17) | 0.0004 |
| Tb.N (1/mm) | 0.95 (± 0.03) | 1.44 (± 0.37) | 0.0025 |
| Ct.Ar/T.Ar (%) | 40.1 (± 1.3) | 45.1 (± 3.23) | 0.002 |
| Ct.Th (mm) | 0.18 (± 0.008) | 0.21 (± 0.02) | 0.003 |
| J (mm^4^) | 0.36 (± 0.04) | 0.41 (± 0.02) | 0.026 |
| Imax (mm^4^) | 0.23 (± 0.03) | 0.26 (± 0.02) | 0.023 |
| Imin (mm^4^) | 0.13 (± 0.01) | 0.145 (± 0.01) | 0.078 |

AVD, apparent volume density; BV/TV, trabecular bone volume fraction; BS/TV, trabecular bone surface density; BS/BV, specific bone surface; Tb.Th, trabecular thickness; Tb.Sp, trabecular separation; Tb.N, trabecular number; Ct.Ar/T.Ar, cortical area fraction; Ct.Th, cortical thickness; J, polar moment of inertia; Imax and Imin, second moment of inertia; *^a^* statistical significance was determined using Student’s *t*-test. All results are expressed as mean ± S.D.
