# Supplementary figures and images for: Role of HTRA1 in bone formation and regeneration: In vitro and in vivo evaluation
Source: PLoS One. 2017 Jul 21;12(7):e0181600. doi: 10.1371/journal.pone.0181600 (PMC5521800; doi:10.1371/journal.pone.0181600)

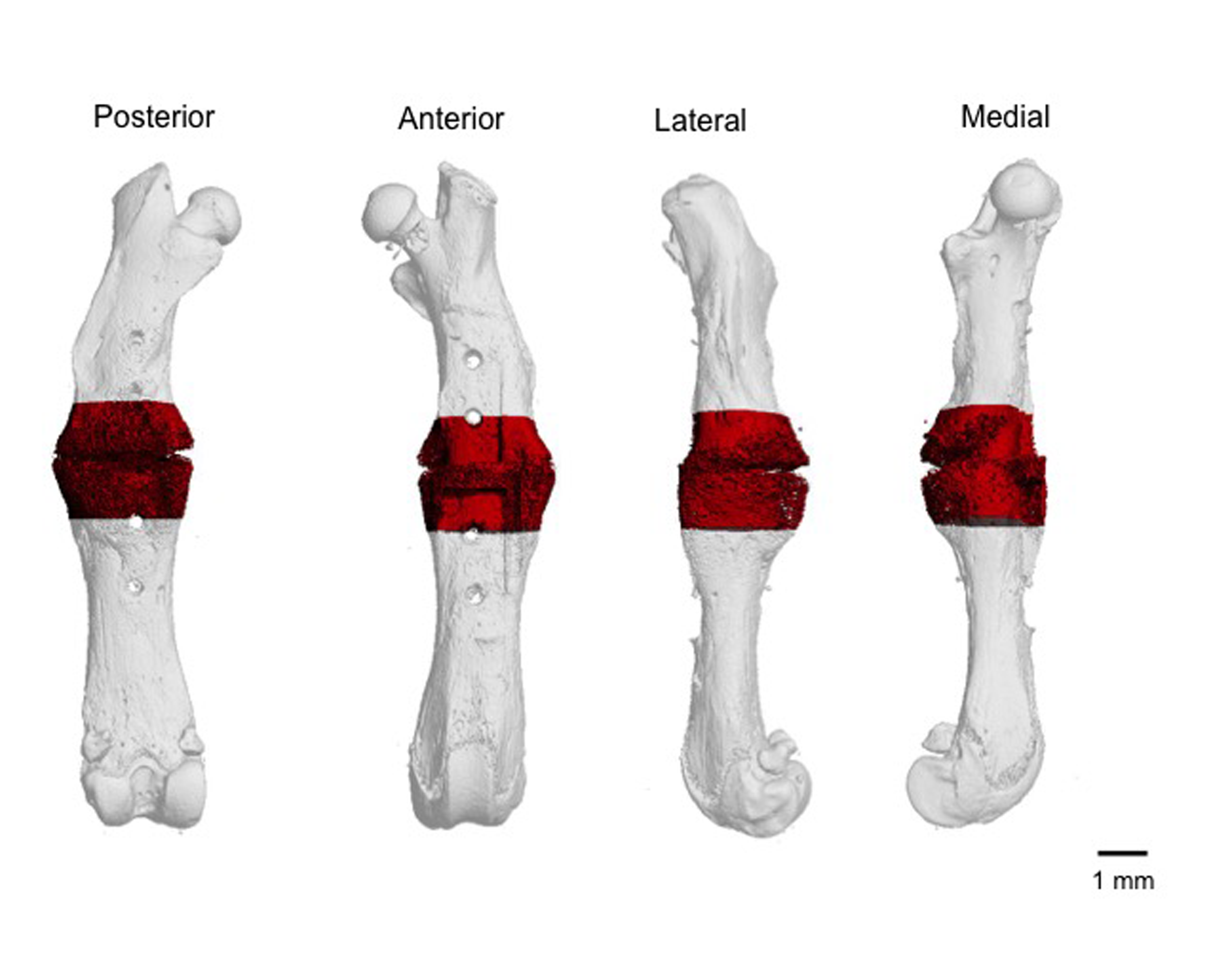

Supplement: S1 Fig — Representative images of posterior, anterior, lateral and medial aspects of an Htra1-KO mouse femur at 21 days following osteotomy. The red colouration highlights the mineralized tissue within the volume of interest (500 x 500 x 280 voxels) as observed following removal of the MouseFix plate. (TIF) [file pone.0181600.s004.tif]

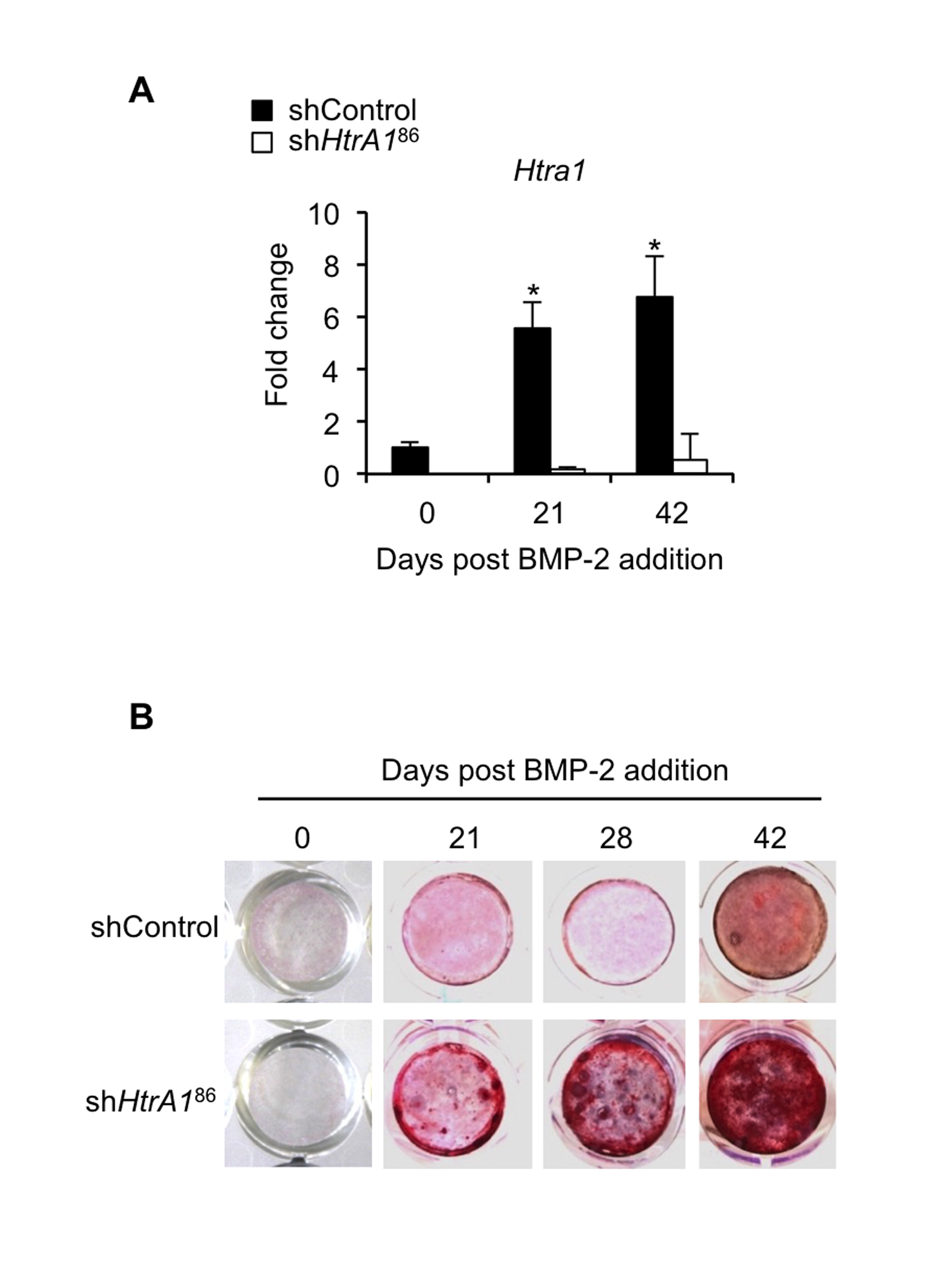

Supplement: S2 Fig — (A) RT-qPCR analysis was used to confirm efficient knockdown of Htra1 gene expression in C3H10T1/2 cells transduced with Htra1-specific shRNA (shHtra186) at selected time points following stimulation with rhBMP-2 (100 ng/ml). Gene expression levels were determined using the 2-ΔΔCT method and presented as fold change relative to uninduced cells at day 0 (value = 1). All values are expressed as mean ± S.D. (triplicates). *P < 0.01 comparison between shControl and shHtra186 using one-way ANOVA. (B) CH310T1/2 cells stably transduced with non-target control shRNA (shControl) or Htra1-specific shRNA (shHtra186) were stimulated with rhBMP-2 (100 ng/ml) for up to 42 days and stained with Alizarin Red S. (TIF) [file pone.0181600.s005.tif]

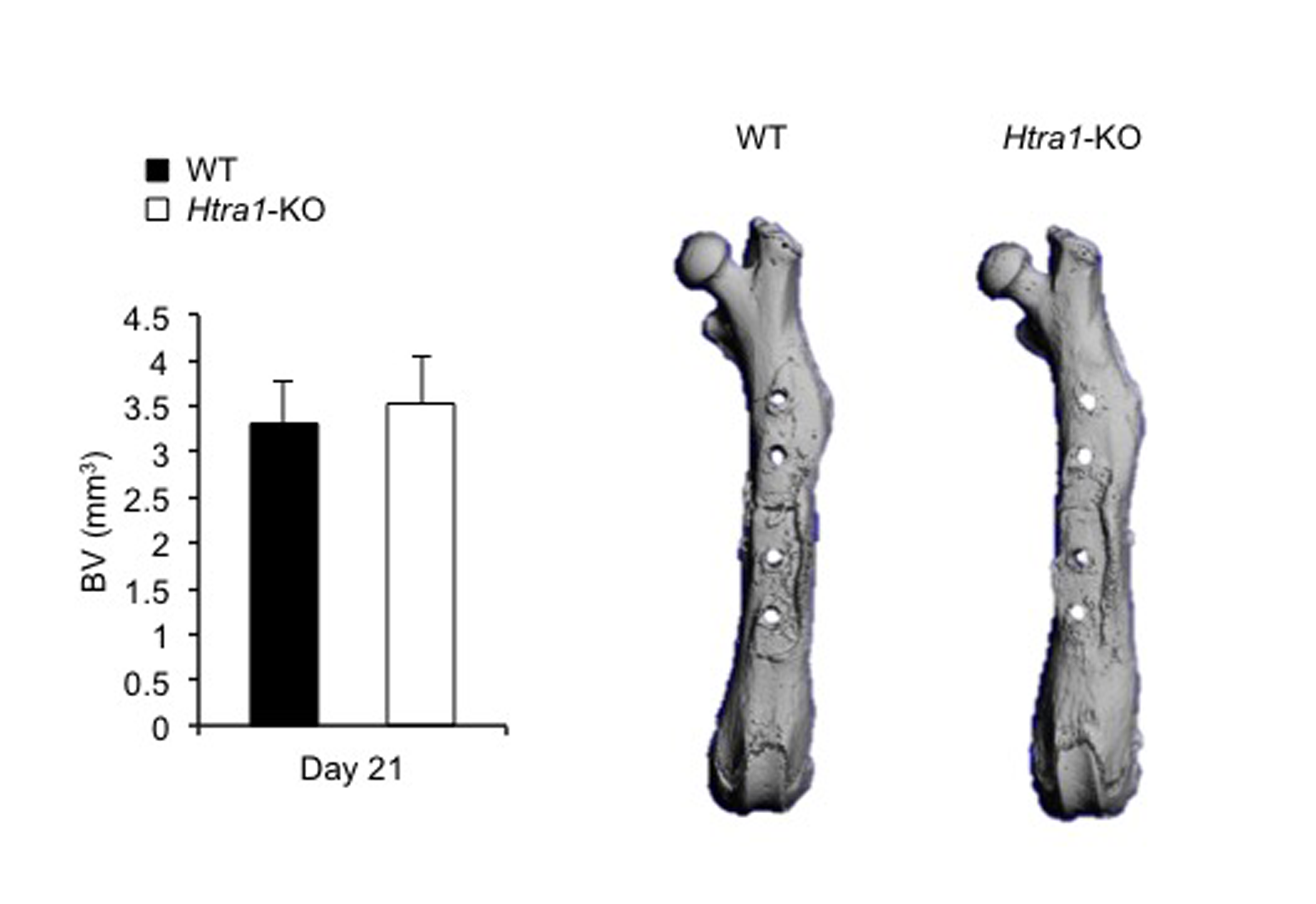

Supplement: S3 Fig — Micro-CT evaluation of bone volume (BV) in osteotomy sites of femurs stabilized with a rigid MouseFix plate from wild-type (WT) (n = 9) and Htra1-knockout (Htra1-KO) (n = 9) mice at 21 days after femoral osteotomy. All values are expressed as mean ± S.D. (TIF) [file pone.0181600.s006.tif]

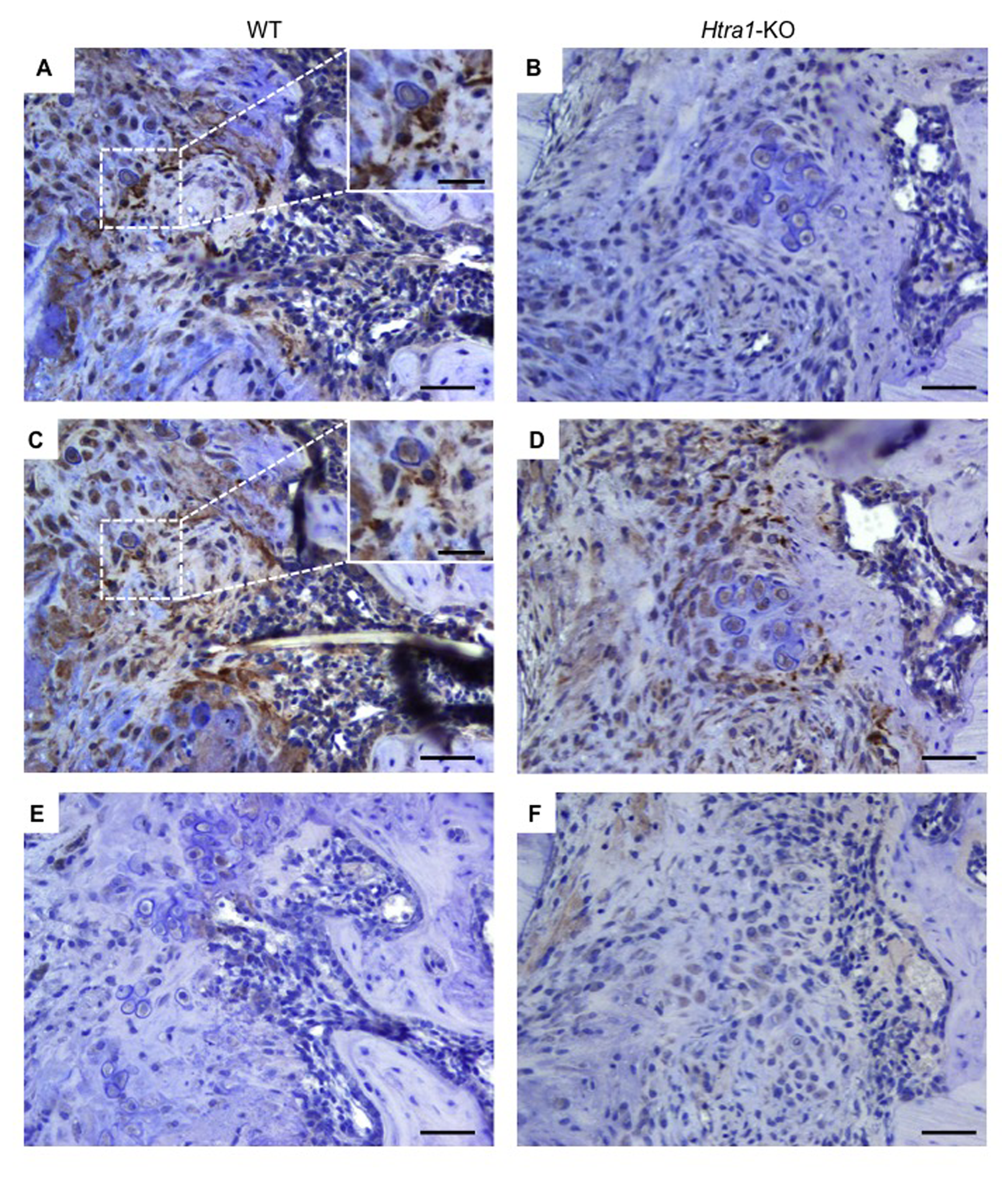

Supplement: S4 Fig — Representative micrographs of anti-HTRA1 (A, B), anti-HTRA3 (C, D), or normal rabbit serum (E, F) stained paraffin wax sections of femurs from WT (A, C, E) and Htra1-KO (B, D, F) mice 21 days after osteotomy. HTRA1 and HTRA3 staining was detected using horseradish peroxidase-diaminobenzidine (brown) and sections counterstained with Harris modified hematoxylin (blue). Main scale bar = 50 μm; inset scale bar = 25 μm. (TIF) [file pone.0181600.s007.tif]

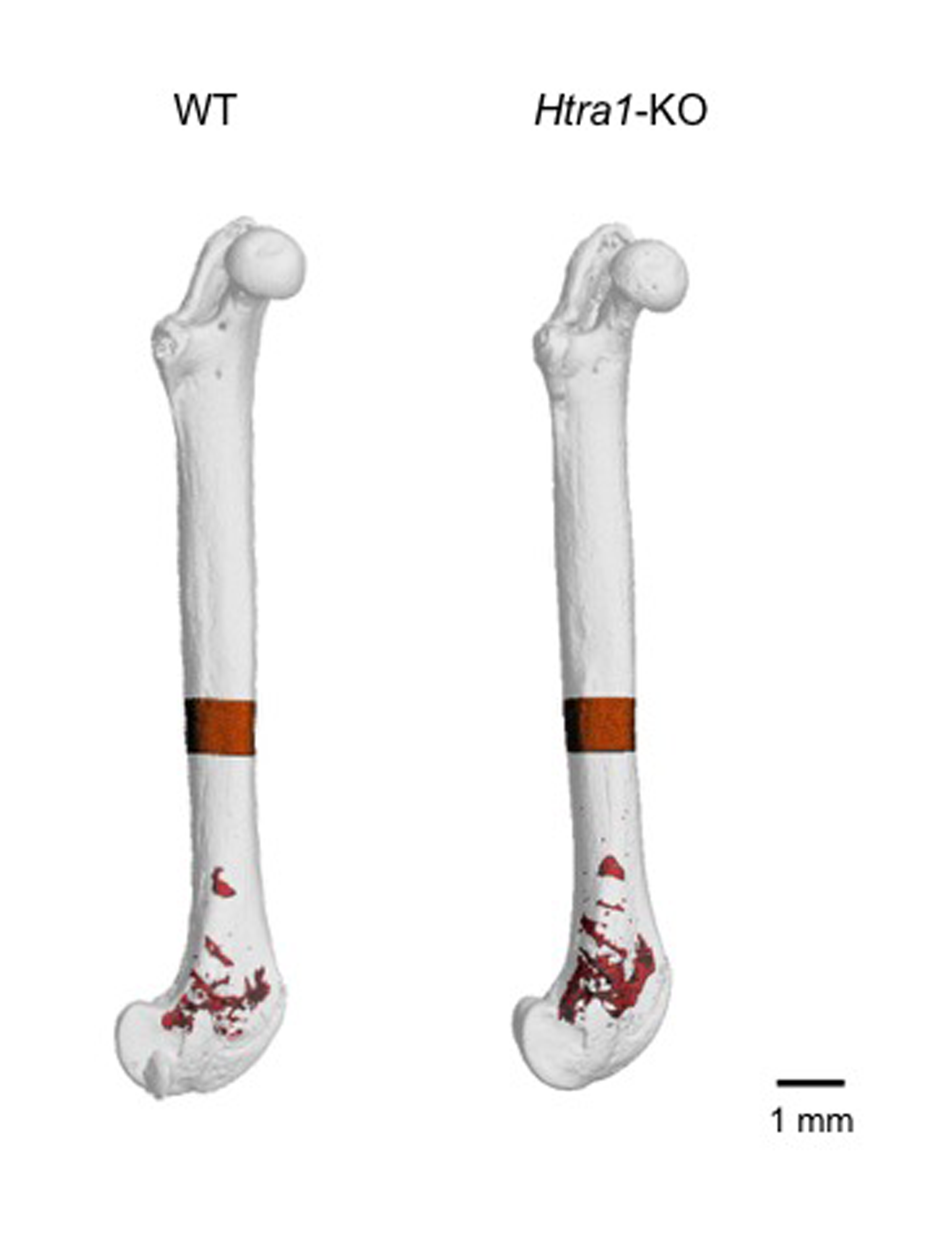

Supplement: S5 Fig — Selected images of distal femurs from wild-type (WT) and Htra1-knockout (Htra1-KO) mice illustrating the regions from which cortical (orange) and trabecular (red) bone measurements were taken. Images are representative of the median trabecular BV/TV value from each group. (TIF) [file pone.0181600.s008.tif]
